# Quality of diet plans for weight loss featured in women’s magazines. A cross-sectional descriptive study: Qualidade de planos dietéticos para perda de peso veiculados em revistas femininas. Um estudo descritivo transversal

**DOI:** 10.1590/1516-3180.2016.0301280217

**Published:** 2017-07-31

**Authors:** Maiara Martinighi, Edina Mariko Koga da Silva

**Affiliations:** I BSc. Nutritionist and Master’s Student in the Postgraduate Program on Evidence-Based Health, Universidade Federal de São Paulo (Unifesp), São Paulo (SP), Brazil.; II MD, PhD. Associate Professor, Discipline of Emergency Medicine and Evidence-Based Health, Universidade Federal de São Paulo (Unifesp), São Paulo (SP), Brazil.

**Keywords:** Nutritive value, Diet, Weight loss, Publications, Female

## Abstract

**CONTEXT AND OBJECTIVE:**

Brazil has the fifth largest population of obese individuals in the world. Women’s magazines publish a large number of diet plans, and therefore the objective of this study was to assess the quality of these plans.

**DESIGN:**

Cross-sectional descriptive study.

**METHODS:**

We included the Brazilian women’s magazines of highest circulation published between January and June 2014 that advertised diets for weight loss on their covers. We extracted the quantities of macro and micronutrients from each of these diet plans and compared these quantities with the World Health Organization nutritional guidelines for adult women. We also checked the total energy quantities of these plans, and any recommendations about water intake and physical activity.

**RESULTS:**

We identified 136 potentially eligible magazine issues; 41 were excluded and 95 issues of 6 different magazines were included in the study. We found that 83.1% of the plans had carbohydrate and fiber levels below the recommendations. On the other hand, the protein and saturated fatty acid levels were above the recommendations in 97.8% and 95.7% of the plans, respectively; 75.7% of the diets had inadequate calcium levels and 70.5% had low iron levels. Only 30 plans specified the total daily quantity of dietary energy and in 53.3% of these, the information was inconsistent with our estimates; 20% of the plans had no recommendations on daily water intake and 37.5% did not give recommendations regarding physical activity practices.

**CONCLUSION:**

The diet plans for weight loss featured in Brazilian women’s magazines are of low quality.

## RESUMO

**CONTEXTO E OBJETIVO:**

O Brasil tem a quinta maior população de obesos do mundo. Revistas femininas publicam um grande número de planos dietéticos. Assim, o objetivo deste estudo foi avaliar a qualidade desses planos.

**MÉTODOS:**

Incluímos as revistas femininas brasileiras de maior circulação publicadas entre janeiro e junho de 2014 que tivessem uma chamada de dieta para perda de peso em sua capa. De cada plano dietético, foi extraída a quantidade de macro e micronutrientes. Comparamos esses valores com as diretrizes nutricionais da Organização Mundial de Saúde para mulheres adultas. Verificamos também a quantidade total de energia, se o plano recomendava consumo de água e prática de atividade física.

**RESULTADOS:**

Um total de 136 exemplares potencialmente elegíveis foi identificado; 41 foram excluídos e 95 exemplares de 6 revistas diferentes foram incluídos no estudo. A análise mostrou que 83,1% dos planos apresentaram valores de carboidratos e de fibras menores do que os recomendados. Por outro lado, o teor de proteína e de ácidos graxos saturados foi maior do que o recomendado em 97,8% e 95,7% dos planos, respectivamente, e 75,7% dos planos tinham teor inadequado de cálcio e 70,5% de ferro. Apenas 30 planos especificaram a quantidade total de energia diária e em 53,3% desses, a informação foi discordante com nossas estimativas; 20% dos planos não recomendavam consumo diário de água, 35,7% não recomendavam a prática de atividade física.

**CONCLUSÃO:**

A qualidade dos planos dietéticos para perda de peso veiculados em revistas femininas brasileiras é baixa.

## INTRODUCTION

There are currently 2.1 billion overweight individuals in the world, which represents a huge increase in relation to the 875 million overweight individuals in the 1980s. Estimates indicate that obesity and overweight caused 3.4 million deaths worldwide in 2010 and reduced life expectancy by 3.9% years.^1^ Brazil is the country with the fifth largest number of obese individuals in the world, surpassed only by the United States, China, India and Russia.^1^ In São Paulo, the largest city in Brazil, 18.2% of all women and 17.5% of all men are obese.^2^ Emotional and genetic factors, along with an overall increase in dietary energy and a sedentary lifestyle, are the main causes of the increased prevalence of obesity.^3^^,^^4^

In parallel with the national increase in the prevalence of obesity, the last three decades stand out as a period during which body image and “being fit” became fundamental values within Brazilian culture. Nowadays, fitness and body image are highly prioritized and a large number of individuals regard any slight weight gain as a major problem. Many people whose weight is within the normal range feel that they are overweight. In this culture, simply accepting one’s body shape is frowned upon since the body is always imperfect and in need of correction or transformation.^5^^,^^6^^,^^7^

Despite the many health consequences and the high economic costs of obesity, the search for an efficient diet for weight loss and maintenance is still ongoing. The World Health Organization (WHO) recommends that, in order to promote healthy and gradual weight loss, diet plans should limit dietary energy consumption and encourage physical activity.^8^^,^^9^

There is relentless pursuit of a fit body that conforms to the standards imposed by society, and constant surveillance of food intake according to recommended diet plans that produce a slow and steady weight loss. These coexist with an avalanche of popular diet plans that promise quick and easy ways to slim down. Most modern popular diets encourage reducing or excluding a specific macronutrient or excessive reduction of total dietary energy, and are only efficient over the short term.^10^^,^^11^^,^^12^

The media plays an important role in providing information about eating habits, nutrition, health and related matters, including diet plans and fitness programs.^12^^,^^13^^,^^14^ Most women are exposed to all kinds of diet plans for weight loss in magazines, which also lead them to be intensely concerned about their weight, appearance and body image. Women of all sizes and shapes are motivated to lose weight because of fashion and not because of the health risks associated with being obese or overweight.^11^ It is also increasingly common to find nutrition articles in popular magazines that include interviews with celebrities. When public figures (especially actors and athletes) give testimonials about their eating habits, these can have far-reaching and potentially harmful effects on the population because many readers tend to believe any information endorsed by role models.^15^

Professional nutritionists can and should also use these media channels to reduce the lack of information and confusion that lay people have about nutrition and to disseminate correct facts about adequate eating habits, to promote health. These educational efforts can also be very useful when working with group or individual nutritional counseling.^15^^,^^16^^,^^17^^,^^18^^,^^19^

In Brazil, women buy 60% of the popular magazines.^20^ Many of these weekly or monthly issues publish a large number of diet plans of unknown quality. Therefore, it is important to assess the content and adequacy of these diet plans from a nutritional perspective. The results from the present study will help to inform the public about the potential harm and benefits of diet plans published in popular magazines.

## OBJECTIVES

To assess the quality of diet plans for weight loss published in Brazilian women’s magazines.

## METHOD

This was a descriptive cross-sectional study conducted in the postgraduate program of the Federal University of São Paulo and approved by the Research Ethics Committee of the institution under the number 25223.

The analysis unit was diet plans for weight loss presented in articles published in popular women’s magazines. The articles included in this study were obtained using a pre-specified sampling strategy. We selected the magazines with the highest circulation between January and June 2014 that were classified as addressing “feminine”, “behavior and beauty” and “quality of life and health” matters, according to the 2014 Brazilian media data registry.^20^^,^^21^ All of the issues of these magazines that advertised diets for weight loss on their covers were considered eligible for inclusion.

After reading each potentially eligible full-text article, in printed versions of the journals, we excluded those that did not provide a full diet plan (i.e. that did not allow us to extract the nutritional content of the plan), those that only provided general nutritional recommendations and those that proposed diet plans lasting less than 7 days.

We extracted data from all the eligible articles using a form specifically created for the study. Estimation of the total caloric values and nutritional composition of the diet plans of each article included was performed in duplicate by two independent investigators using the DIETPRÓ software, the database of the AVANUTRI software and the Brazilian Food Composition Table.^22^

We analyzed the adequacy of each plan suggested in the magazine articles by comparing the estimated nutritional macro and micronutrient content of the diet plan with international nutritional recommendations for women between 19 and 50 years of age. For carbohydrates, proteins, fats and saturated fatty acids, we compared the diet plan content according to the latest WHO recommendations.^23^ Briefly, these recommend that adults should get 55% to 75% of their total daily calories from carbohydrates, 15% to 30% from fats (less than 10% from saturated fatty acids) and 10% to 15% from proteins. We classified diet plans with less than 55% carbohydrates as being below the recommendations, those between 55% and 75% as adequate and those with more than 75% carbohydrates as above the recommendations. The fat content was categorized as low when it represented less than 15% of the total dietary energy, adequate when it was between 15% and 30% and high when it was more than 30%. Plans with saturated fatty acid values below 10% were considered adequate and above this were considered high. Plans in which the protein content accounted for between 10% and 15% of the total dietary energy were classified as being adequate; those below and above these figures were categorized as being below and above the recommendations, respectively.^23^^,^^24^

We assessed the fiber content of each diet according to the latest dietary reference intake (DRI) values proposed by the Institute of Medicine (IOM).^25^ Diet plans with less than 25 g of fibers were categorized as having low fiber content and those with 25 g or more were considered adequate, since there is no upper reference level for fibers.

We analyzed the micronutrient content (calcium, iron, vitamin C and sodium) of the diet plan according to the IOM recommendations for vitamins and minerals.^26^^,^^27^^,^^28^^,^^29^ For calcium, we used the micronutrient apparent adequacy criterion proposed by the DRI.^30^ The formula used was: Z = Y - EAR/√Vnec + (Vint/n), where Y = average intake of micronutrients obtained from food surveys, or in this case, the diet plans of women’s magazines; EAR = estimated average requirement, i.e. mean micronutrient needs according to age and sex; Vnec = variance of needs, which corresponds to 10% of EAR = 0.1 x EAR; and Vint = intrapersonal variance, according to age and sex. Intrapersonal standard deviation (SD) was obtained.^30^

We analyzed the vitamin C and iron content, as suggested by the IOM subcommittee that published the DRI.^30^^,^^31^ Values below the estimated average requirement (EAR), which is 60 mg for vitamin C and 8.1 mg for iron, were considered to be lower than the recommendations. Values between the EAR and the recommended dietary allowances (RDA), which are 75 mg and 18 mg for vitamin C and iron, respectively, were considered to represent a risk of inadequacy. Values between the RDA and the tolerable upper intake level (UL), which is 2000 mg for vitamin C and 45 mg for iron, were considered appropriate and values above the UL were considered to be higher than the recommendations.^30^^,^^31^ For sodium, the adequate intake and UL values were used, since there is no EAR value for this mineral. Values between the adequate intake and UL were considered appropriate and values greater than the UL were considered to be higher than the recommendations.^28^

We also checked whether the article specified the total daily calorie content of the diet plan, and whether it recommended appropriate daily water intake and encouraged readers to practice physical activities along with the diet plan. We calculated the total daily calorie content of each plan and compared this with the values provided in the articles that presented this information. The total dietary energy of the diet plan was considered correct if it was between 90% and 110% of our calculations.^18^^,^^19^^,^^32^ Finally, we checked whether the article cited the name of the professional responsible for elaboration of the plan.

The results are presented using descriptive statistics.

## RESULTS

**Figure 1.**
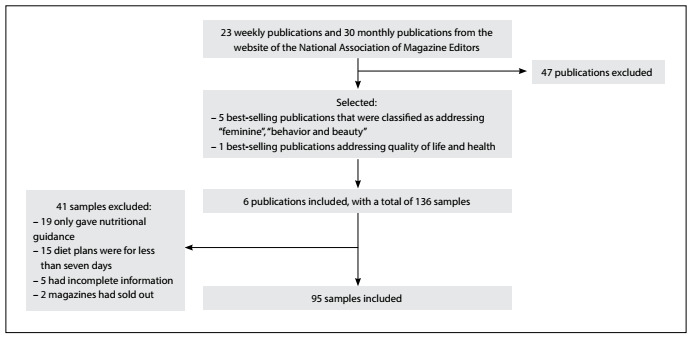
Flowchart of the sample acquisition: January-June 2014.

We identified a total of 53 different women’s magazines (23 weekly and 30 monthly titles) on the website of the National Association of Magazine Editors. Six different women’s magazines (five weekly and one monthly) were included in the study. The numbers of copies sold and types of the magazines were: 188,895 (A, weekly); 137,138 (B, weekly); 125,774 (C, weekly); 67,500 (D, weekly); 27,520 (E, weekly); and 209,772 (F, monthly). A total of 136 issues of these six magazines had diet plans on their covers and were selected for full-text reading; 41 were excluded for various reasons and 95 articles were included in the study (Figure 1).

The 95 articles included were published in the six different magazines as follows: 11 articles (11.5%) in magazine A; 21 (22.1%) in magazine B; 17 (17.8%) in magazine C; 25 (26.3%) in magazine D; 15 (15.7%) in magazine E; and 6 (6.3%) in magazine F.

Quantitative analysis on the content of the 95 diet plans revealed that 79 plans (83.1%) had carbohydrate levels and 93 (97.8%) had protein levels below those recommended by the reference used. On the other hand, 63 (66.3%) of the plans proposed adequate fat levels and 91 (95.7%) of the plans had saturated fatty acid levels above the recommendations (Table 1).

**Table 1.**
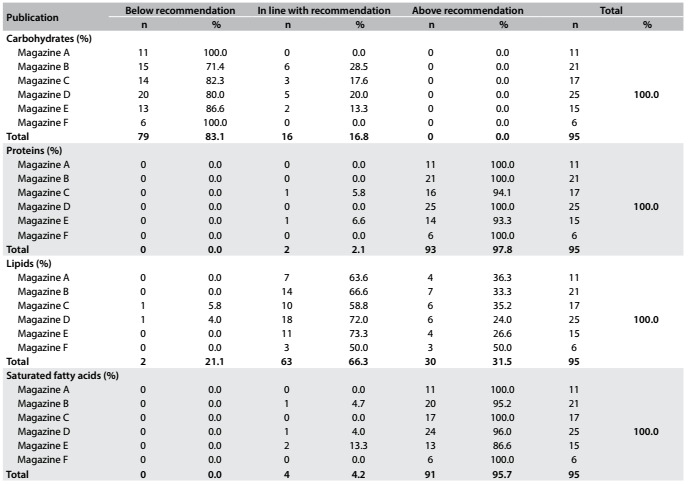
Quantitative analysis on the adequacy of provision for carbohydrates, proteins, lipids and saturated fatty acids.

Seventy-nine (83.1%) of the plans had a fiber content lower than the recommendations, but 49 (51.5%) and 85 (89.4%) of the plans had adequate sodium and vitamin C levels, respectively. Sixty-seven (70.5%) of the plans were categorized as having an iron level that represented a risk of inadequacy (Tables 2 and 3). Finally, 72 (75.7%) of the plans had a 70% to 98% likelihood of inadequacy in relation to calcium.

**Table 2.**
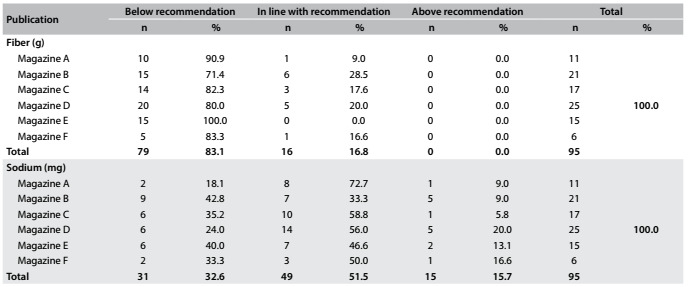
Quantitative analysis on the adequacy of provision for fiber and sodium.

**Table 3.**
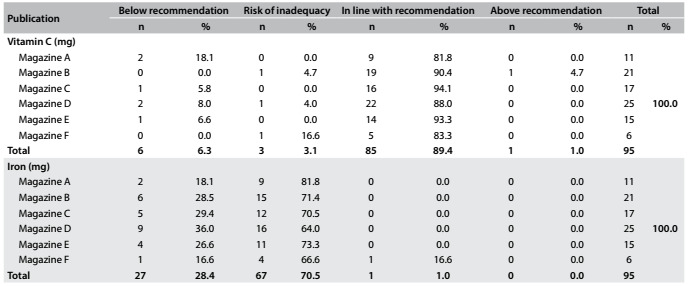
Quantitative analysis on the adequacy of provision for vitamin C and iron.

Less than one third (n = 30; 31.5%) of the diet plans specified the total dietary energy expressed in calories. In 16 (53.3%) of these plans, the total informed by the magazine was discordant with our calculations: in 9 plans (56.2%), our calculation was more than 10% higher than the number specified in the magazine; and in 7 (43.75%), our calculation was more than 10% lower than this number. Only 19 (20%) of the articles provided information on the daily water intake.

Approximately one third (n = 34; 35.7%) of the articles encouraged readers to increase their level of physical activity along with the diet for weight loss.

Almost all (n = 92; 96.8%) of the articles stated the name of the professional responsible for elaborating the diet plan or who provided advice or comments about it. In most cases (n = 84; 88.4%), this professional was a nutritionist; in 6 articles (6.3%), a physician was named, and in 2 (2.1%), a phytotherapist was named. Three (3.1%) of the diet plans did not present any health professional in charge. However, one of these (33.3%) was based on the method for losing weight proposed by the French nutrologist Pierre Dukan and one (33.3%) was based on the method of the American physician Ian Smith.

## DISCUSSION

In view of the large number of women’s magazines that publish diet plans for weight loss, the results from this study raise concerns about the potential impacts on health from following these plans. Less than 20% of the 95 plans proposed diets with adequate carbohydrate content, less than 5% presented adequate saturated fatty acid content and less than 3% proposed diets with appropriate protein intake, according to WHO standards.^23^^,^^24^

Simple exclusion of food sources of carbohydrates such as fruits, vegetables and grains from an individual’s usual diet will typically lead to a calorie deficit of approximately 500 calories per day, thus resulting in a loss of 0.45 to 0.9 kg per week. In turn, diet plans that reduce or restrict carbohydrate consumption lead to a loss of 2 to 3 kg in the first week.^10^ This extra loss is not due to a change in metabolism, which would lead to an increase in lipolysis, but to increased diuresis induced by the diet. Even by increasing the 24-hour energy expenditure from 2% to 3%, this effect is responsible for only a small fraction of the weight loss.^10^^,^^33^ Because low carbohydrate diet plans restrict consumption of fruit and vegetables, they are notoriously deficient in micronutrients.^33^

A systematic review compared the effects of low carbohydrate/high protein (LC/HP) versus low fat/high carbohydrate (LF/HC) diet plans on weight loss. At six months, there were weight reduction of up to 4.02 kg in favor of the LC/HP plan (P < 0.00001); but after 12 months, this difference dropped to 1.05 kg (P < 0.05). Evidence from this systematic review shows that LC/HP diets are more effective than LF/HC diets for weight reduction at 6 months, but their effectiveness decreases by the 12^th^ month.^34^

A prospective Japanese study involving two cohorts that were followed for 9 to 14 years tested the hypothesis that the intake of saturated fatty acids is inversely associated with the risk of stroke and directly associated with coronary heart disease. The investigators reported that there was a direct association between saturated fatty acid intake and myocardial infarction, mainly among men. On the other hand, they found an inverse association between saturated fatty acid intake and ischemia.^35^

Only 16.8% of the 95 diet plans provided fiber content within the DRI recommendations.^25^ Fibers have important physiological effects, including prevention of intestinal diseases, treatment of obesity and reduction of serum lipid levels.^11^^,^^36^^,^^37^^,^^38^^,^^39^ From a meta-analysis on 67 randomized trials assessing the effect of dietary fiber on serum cholesterol levels, it was found that the consumption of 2-10 g of soluble fibers per day was associated with a significant reduction in total cholesterol levels.^38^

Almost 70% of the diet plans did not specify the total daily quantity of calories, and in over half of those that provided this information, it was incorrect. All diet plans that propose reductions in total daily calorie intake will lead to weight loss. In the absence of physical activity, a plan that provides between 1400 to 1500 calories per day, regardless of the macronutrient percentage, will result in weight loss.^40^

A previous Brazilian study published in 2004 also assessed diet plans for weight loss that were featured in non-scientific publications. The authors reported that 80% of the diet plans did not provide any information on daily intake of water and less than 25% had appropriate macronutrient distributions. In fact, all of the plans had inadequate protein content, with one third of them recommending that over 15% of the total calorie intake should come from that macronutrient. Moreover, 86% of those plans provided inappropriate calcium content, 92% gave rise to inadequate vitamin E content and 97% did not provide adequate iron content.^12^ In our study, carried out ten years later, 20% of the diet plans did not provide any information on daily intake of water and less than 4% had appropriate macronutrient distributions. In relation to protein, 97.8% of them recommended that over 15% of the total calorie intake should come from that macronutrient. The vitamin E content of the diet plans was not verified in this study, but 75.7% of the plans presented a probability of being inadequate in relation to calcium and 70.5% did not provide adequate iron content.

Less than 13% of the diet plans provided adequate calcium content. Calcium is an essential mineral at different stages of women’s lives, especially with regard to maintaining bone health.^26^^,^^41^ Consumption of high protein diets, especially for long periods, may cause increased urinary calcium loss, thus increasing the risks of osteoporosis.^42^^,^^43^ A prospective Norwegian study reported that there was a significantly higher risk of hip fracture among women with high intake of non-dairy animal protein and low calcium intake (RR 1.96; 95% CI 1.09-3.56).^44^

The lack of recommendations about water intake in most diet plans for weight loss that are available in popular women’s magazines is worrying. Under normal environmental conditions and energy expenditure, an average adult will need approximately 1 ml of water per calorie. The need will be higher among individuals who have an exercise routine and among those who are on a protein-rich diet. Deficiency in water intake manifests quickly and a change in the body’s water content as small as 1% promptly causes symptoms of dehydration.^45^

Almost two thirds of the plans included in our study did not encourage physical activity along with the diet, despite the well-known fact that exercise is an important part of any weight loss and maintenance program.^44^^,^^46^^,^^47^

Although our study did not evaluate the readers’ actual use of the diets or the possible impact of the dietary plans on their health, it clearly shows that most diets published in popular Brazilian women’s magazines are inadequate. Our results are important for informing the general public about the risks of blindly following the diets published in these magazines and to alert journalists and editors of these publications about the need to check the scientific accuracy and safety of these diet plans before disseminating them to a wide lay audience.

## CONCLUSION

All of the 95 diet plans for weight loss published during a six-month period in popular Brazilian women’s magazines failed to follow one or more of the international dietary recommendations. In this light, the results obtained here emphasize that publication of diet plans for weight loss in non-scientific magazines needs to be based on international dietary recommendations and evidence from studies of good methodological quality. Following these diets may cause deleterious effects to health.
